# *Curcuma longa*-Based Optical Sensor for Hydrochloric Acid and Ammonia Vapor Detection

**DOI:** 10.3390/s23125602

**Published:** 2023-06-15

**Authors:** A. Sánchez Juárez, Fabián Carrión, Javier Carrión, Darwin Castillo, J. P. Padilla-Martínez, Ángel Cruz-Félix

**Affiliations:** 1 Departamento de Química, Universidad Técnica Particular de Loja, Loja 1101608, Ecuador; ffcarriont@gmail.com (F.C.); jfcarrion1@utpl.edu.ec (J.C.); 2 Instituto de Ciencias, Benemérita Universidad Autónoma de Puebla, Puebla 72960, Mexico; juan.padilla@correo.buap.mx; 3 Departamento de Óptica, Instituto Nacional de Astrofísica, Óptica y Electrónica, Puebla 72840, Mexico; sinue@inaoep.mx

**Keywords:** gas sensors, optical fiber, cumin, turmeric, industrial safety, applied optics, chlorohydric acid, ammonia

## Abstract

In this research, we present a prototype optical system that offers significant advances in detecting hydrochloric acid (HCl) and ammonia (NH3) vapors. The system utilizes a natural pigment sensor based on *Curcuma longa* that is securely attached to a glass surface support. Through extensive development and testing with HCl (37% aqueous solution) and NH_3_ (29% aqueous solution) solutions, we have successfully demonstrated the effectiveness of our sensor. To facilitate the detection process, we have developed an injection system that exposes *C. longa* pigment films to the targeted vapors. The interaction between the vapors and the pigment films triggers a distinct color change, which is then analyzed by the detection system. By capturing the transmission spectra of the pigment film, our system allows a precise comparison of these spectra at different concentrations of the vapors. Our proposed sensor exhibits remarkable sensitivity, allowing the detection of HCl at a concentration of 0.009 ppm using only 100 µL (2.3 mg) of pigment film. In addition, it can detect NH_3_ at a concentration of 0.03 ppm with a 400 µL (9.2 mg) pigment film. Integrating *C. longa* as a natural pigment sensor in an optical system opens up new possibilities for detecting hazardous gases. The simplicity and efficiency of our system, combined with its sensitivity, make it an attractive tool in environmental monitoring and industrial safety applications.

## 1. Introduction

Fiber optic applications are changing how we live, mainly how we produce and develop industry. Fiber optics can be used in various multipurpose sensors [1], from gas sensing [2,3], to displacement sensors, to pH and viscosity sensors in different configurations and optical principal effects [4,5,6,7,8,9]. In 1975, fiber optic sensing was introduced for various purposes, and within a few years, the technology was used in oil extraction and medical purposes. Over the years, different gas detection methods have been introduced, as the monitoring and early detection of harmful compounds concern scientists and field workers [10,11]. Cordero et al. [12] detected chlorine and hydrogen sulfide by coating the optical fiber with indicators, fluorinated polymers, and silica. A 10 m long fiber could detect from 1 ppm to 20 ppm of chlorine and hydrogen sulfide. The sensor was expected to be fully operational for 1–2 years. Fiber optic gas sensors are also possible using inorganic compounds [13,14]. It is possible to detect benzene, acetone, and isopropyl alcohol vapors by coating an optical fiber with nanocrystalline zinc oxide. The detection limits of this type of sensor can range from 0 to 500 ppm of gas, and it can fully recover after 42 min of exposure to the gas [15]. Organic compounds may also be suitable for gas detection, providing a more versatile and environmentally friendly sensor.

A nanoporous matrix of polylactic acid was effective in the adsorption and diffusion of HCl gas. This matrix, containing 5,10,15,20-tetraphenyl porphyrin, showed protonation upon exposure to the gas, resulting in a color change from pink to green. The sensor achieved exceptional detection performance, with a lower detection limit of 34 ppb and a response time of 5 s. Due to its complete reversibility, the sensor could be tested up to ten times using the same nanoporous array [16]. The applications of optical fibers and microfibers for sensing various parameters show different techniques, from very simple to more complex [17,18,19,20,21,22,23].

In this research, a fiber optic sensor for the detection of hydrogen chloride (HCl) and ammonia gas (NH_3_) is presented using *C. longa* film as a sensing medium; this film contains curcumin, the active ingredient and principal constituent of turmeric root, which is responsible for its intense yellow-orange color. A detection cell of the injection system was also set up. The cell contains turmeric films, and is simultaneously attached to a light source and an optical fiber to guide the optical response to the spectrometer. Finally, the spectral responses were analyzed to determine the optical detection limits of 0.009 ppm and 0.03 ppm of HCl and NH_3_, respectively. The main advantages of the *C. longa* film-based sensor are the high sensitivity, which allows for easy detection; fast optical response, necessary for this type of gas; good optical stability, essential for industrial applications with variable temperature and humidity; and high ease of sensor replacement, if the film is damaged or has simply expired, one only needs to change the substrate containing the film without changing the optical system.

## 2. Materials and Methods

### 2.1. Extraction and Testing of C. longa Pigment

Commercially available turmeric powder was used to obtain an ethanolic extract to test the response of curcuminoids to HCl and NH_3_. A total of 20 g of turmeric powder was weighed in an analytical balance (Radwag^®^–PS 1000.R2, Radom, Poland) and then transferred to a 1 L amber glass bottle. The bottle was first thoroughly cleaned with a 5:1 ethanol-acetone solution, rinsed with distilled water, and dried with compressed air; 500 mL of absolute ethanol was added to the powder in the bottle. The bottle was shaken vigorously for about 5 min. The bottle was sealed with aluminum foil and parafilm sheets. After labeling, maceration was performed for 7 days with vigorous shaking every 12 h. The contents of the bottle were filtered by gravity into an Erlenmeyer flask with a glass funnel and double filter paper. The volume of turmeric extract obtained was 496 mL. This content was distilled under vacuum (Heidolph Hei-VAP Value, Jinan, China) at 27 °C and 50 rpm rotation. The weight of the dry extract after this procedure was 0.573 g. It is important to note that curcuminoids do not make up the entire extract, but 97%, corresponding to curcumin (C, 77%), desmethoxycurcumin (DMC, 17%), and bisdemethoxycurcumin (BDMC, 3%) [24], the remaining content being solids and minor organic material. To compensate for this difference in extract composition, we calculate the total yield (Y_T_) of the extraction based on the curcuminoid content, resulting in a work of 2.779% (Equation (1)).

(1) $$Y_{T}=\frac{Curcuminoids\;content}{Initial\;turmeric\;powder}\left(100\%\right)=\frac{\left(0.97\right)\left(0.573g\right)}{20g}\left(100\%\right)=2.779\%$$

The dry extract was mixed with 25 mL of absolute ethanol, reserved, and labeled as the final pigment to obtain a suitable dye. 10 mL of this final product was transferred to a test tube to measure its approximate density by relating the weight of the pigment to the volume taken. The density of the dye was 0.8112 g/cm^3^, containing 22.9 mg of curcuminoids per 1 mL of ethanol (C = 22.9 mg/mL). The pigment was centrifuged at 600 rpm for 10 min (Unico Power Spin™ C818 VX, Dayton, NJ, USA) to ensure the separation of the residue. The tube for the final pigment composition was washed with a 5:1 ethanol-acetone mixture and labeled “Turmeric extract 25 mL”.

Glass substrates were prepared to deposit the turmeric powder to obtain thin test films.

**Figure 1:**
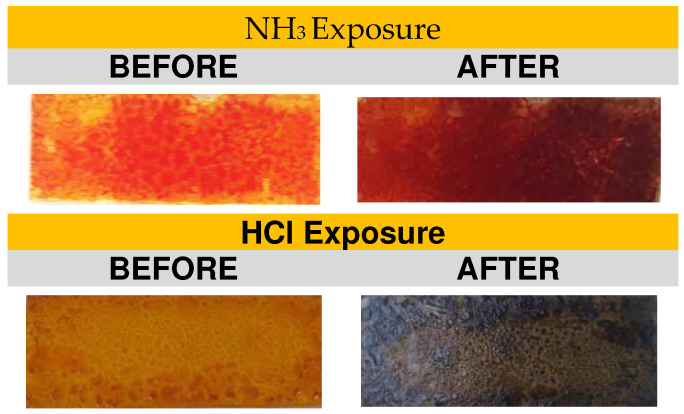
Change in the exposure of turmeric films to NH_3_ and HCl.

Figure 1 shows 2 × 1 cm glass substrates with the pigment thin films. The films were placed in a test cell saturated with NH_3_ vapors for 1 min. The changes were observed, and the films were exposed to air to observe their behavior for 24 h. The HCl vapor test was performed similarly to the NH_3_ test with new pigment films.

For the following evaluation, 16 pigment films were prepared. The pigment was applied to each film using a 100 µL micropipette and air-dried for 15–30 min. Four films containing 100 µL or 2.3 mg of curcuminoids, four films containing 200 µL or 4.6 mg, four films containing 300 µL or 6.9 mg, and four films containing 400 µL or 9.2 mg of curcuminoids were prepared. The amount of curcuminoids in each film was calculated based on the curcuminoid concentration of the pigment.

### 2.2. Sensor Cell Design and Construction

**Figure 2:**
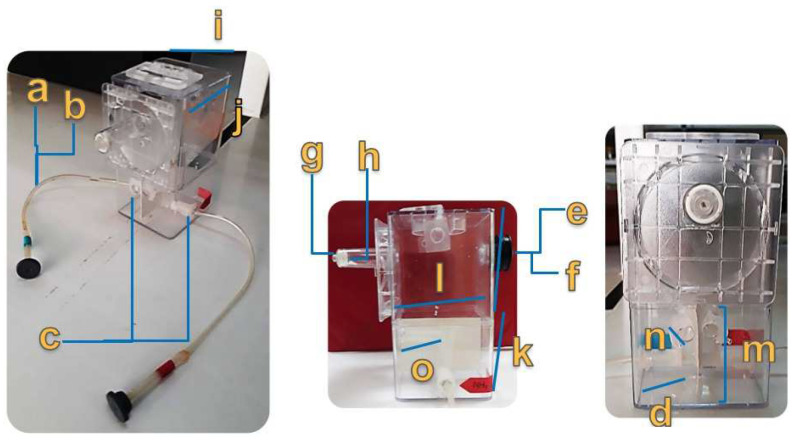
Detailed parts of test cell: (a) injection hose length: 26 cm, (b) injection hose internal diameter: 0.4 cm, (c) injection hose inlet hole: 0.8 cm, (d) injection channels length: 2.5 cm, (e) air inlet diameter: 1.5 cm, (f) air inlet length: 1.5 cm, (g) air outlet: D1 = 0.5 cm; D2 = 1.4 cm, (h) air outlet length: 5.2 cm, (i) major cell width: 7.3 cm, (j) major cell length: 7.3 cm, (k) division height: up = 7.5 cm; down = 5.9 cm, (l) white cardboard length: 4.5 cm, (m) white cardboard height: 7 cm, (n) fiber optic coupling diameter: 0.5 cm, and (o) fiber optic coupling length: 3.5 cm.

A methacrylate box was used as the primary cell. Two plastic spectrophotometric cells were mounted inside the box. Holes were drilled from the box to the smaller cells, and then 0.8 cm diameter plastic tubing was attached from the holes in the cells to the holes in the smaller cells. A syringe tubing was inserted into the channels as an injection line, with one end attached to the inside of the smaller cells and the other attached to an injection septum. One of the cells was used for the HCl test (blue label) and the other for the NH_3_ test (red label). Two additional holes were drilled in the box to attach an optical fiber to the smaller cells for analysis. The methacrylate box was vented at the top, and the smaller cells were separated with white cardboard to avoid interference from the light source. The detailed dimensions of the cell parts are shown in Figure 2.

**Figure 3:**
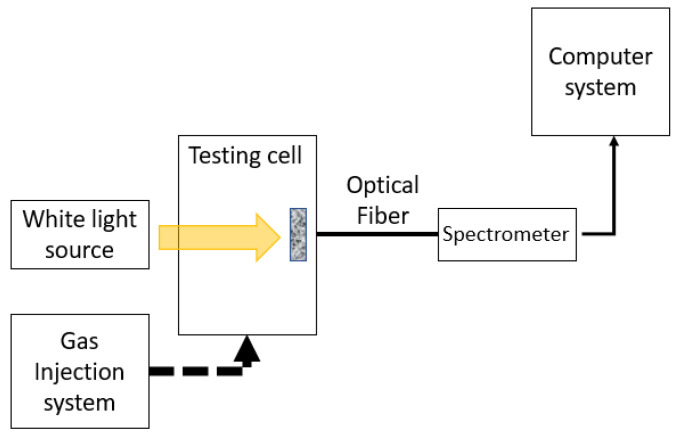
Experimental setup in a controlled gas concentration environment.

A light source (Thorlabs SLS201/M, Newton, NJ, USA) was placed at the back of the cell on the opposite side of the fiber coupling site. A 200 µm, 0.22 NA optical fiber (Thorlabs FT030, Newton, NJ, USA) was coupled to the cell on one side and to the compact spectrophotometer (Thorlabs CCS200/M, Newton, NJ, USA) on the other. Thorlabs OSA 2.9 software was used to quantify and plot the measurements. Figure 3 shows the complete detection system used to perform the experiments on pigment films exposed to NH_3_ and HCl vapors from their respective aqueous solutions.

An initial test was performed on a 100 µL pigment film. A total of 200 µL of NH_3_ solution (29%, Fisher Scientific, Hampton, NH, USA) was injected into the inlet septum of the red section, and the software was set to “sweep”. Thirty readings were taken at 5 s intervals from the injection time, resulting in a total reading time of 150 s.

As observed in the experimental configuration (see Figure 3), inside the test cell was the glass substrate with the turmeric dye film, which gradually changed color when interacting with HCl gas or NH_3_ gas; this color change was transmitted to the spectrometer via fiber optics and analyzed on a computer system.

### 2.3. Main HCl y NH_3_ Exposure Experiments and Data Collection

The lid of the primary cell was opened, and a 100 µL pigment film was placed in the spectrophotometric cell corresponding to the NH_3_ test section (red). The primary cell was closed, and the light source was turned on. A total of 100 µL of NH_3_ solution (29%) was injected into the septum with a 1 mL syringe. The injected contents were pushed with air to the inside of the cell (bottom, where the film was placed) with a 5 mL syringe.

**Figure 4:**
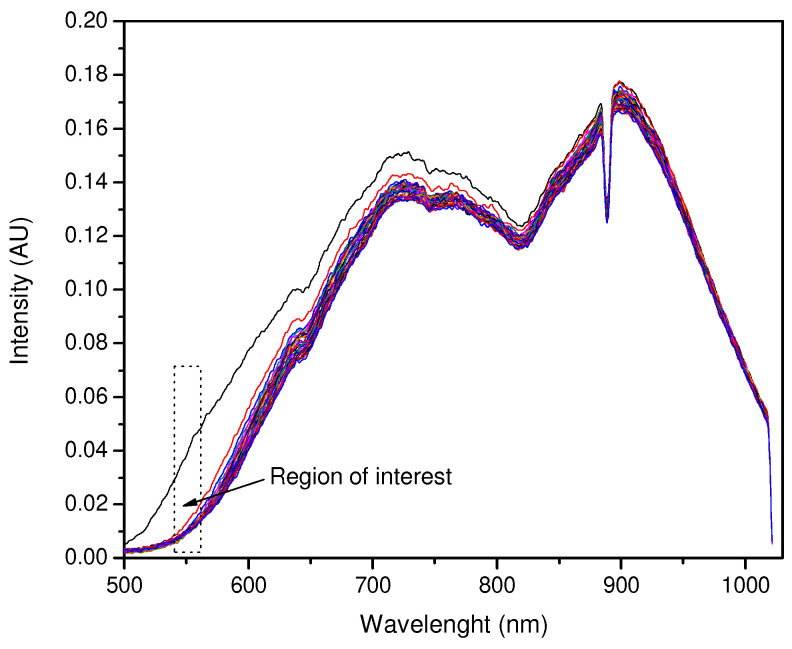
Changes in a 400 µL pigment film to 100 µL of NH_3_. The top black line corresponds to the signal at t = 0, and the remaining lines correspond to 30 lectures.

Readings (see Figure 4) were taken as soon as the NH_3_ solution entered the cell. As in the previous procedure, 30 measurements were taken with a 5 s interval between each reading and a total time of 150 s. The remaining experiments were performed the same way; each pigment film, 100, 200, 300, and 400 µL of pigment were exposed to 100, 200, 300, and 400 µL of NH3 solution, resulting in 16 NH_3_ exposure experiments. The dips in the spectrum around 890 nm are characteristic of the lamp and irrelevant to the spectral analysis.

The pigment films exposed to NH_3_ were allowed to return to their initial state for 24 h. Between each experiment, the cell was ventilated with compressed air through the air inlet for 20 min.

The HCl exposure experiments followed the same procedure as the NH_3_ experiments. Sixteen films were tested with different volumes of HCl solution (37%) using new injection and air-push syringes. After each experiment, the syringes were washed with distilled water to prevent the HCl from reacting with the metal needle. Figure 4 shows the change in wavelength and light intensity due to the color change in the films from orange to dark brown/black.

### 2.4. Evaporation Rate

The evaporation rates of the HCl and NH_3_ solutions were estimated so as to know how many molecules of these gases are required for the sensor to respond and to calculate their detection limits.

The amber vials containing the HCl and NH_3_ were opened and weighed on an analytical balance for 5 min. The vials were then emptied, washed, and dried to obtain their uncapped empty weights. Equations (2) and (3) show the initial and final masses of the solutions and their estimated evaporation rates.

(2) $$R_{NH3}=\frac{\left(42.147-42.119\right)g}{5\;min}=\frac{0.028\;g}{5\;min}=5.6\frac{mg}{min}$$

(3) $$R_{HCl}=\frac{\left(44.290-44.280\right)g}{5\;min}=\frac{0.01\;g}{5\;min}=2\frac{mg}{min}$$

## 3. Results Discussion

### 3.1. Curcumin Light Absorption and pH Interaction

**Figure 5:**
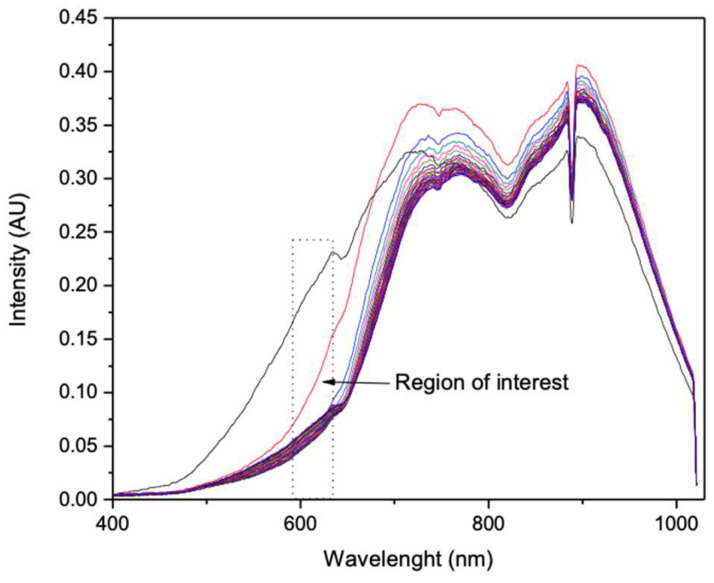
Changes in a pigment film of 100 µL in 100 µL of HCl. The top black line corresponds to the signal at t = 0, and the remaining lines correspond to 30 lectures.

**Figure 6:**
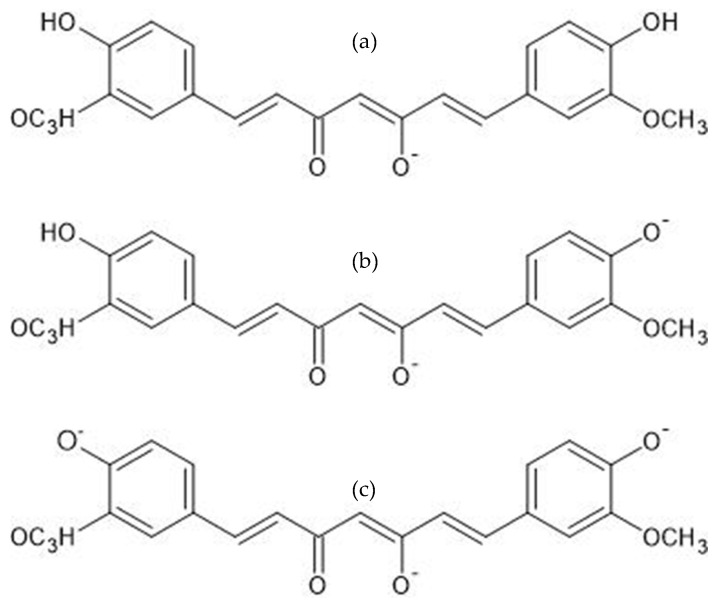
Deprotonated states of curcumin at (**a**) pH7.8, (**b**) pH 8.5 and (**c**) pH 9.

**Figure 7:**
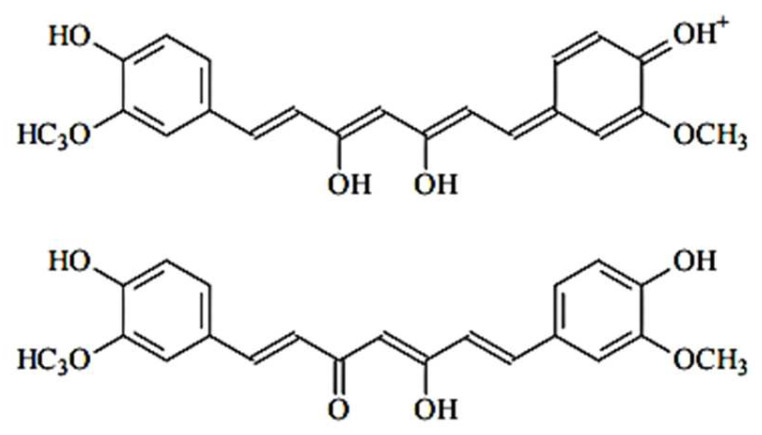
Curcumin protonated states at pH 1–7.

The color changes observed in the pigment films were as follows: from orange to dark red when exposed to NH_3_, and from orange to dark brown/black when exposed to HCl. Both solutions have strong pH values, 1 for HCl and 11 for NH_3_. We assume there is an interaction between the curcuminoids and the free H+ and/or OH- in the solution. It has been shown that the curcumin molecule has two aromatic rings with OH groups, which can be easily protonated or deprotonated, thus acquiring a positive or negative charge, respectively. This change also means the molecule will absorb light differently from its initial state. In an alkaline environment, the acidic protons of the OH group dissociate [25], causing the molecule to absorb light around 620 nm and exhibit a deep red color (Figure 5). In an acidic environment, the molecule gains positively charged protons (Figure 6 and Figure 7). This causes it to absorb light around 680 nm and show a dark brown or black color, indicating that the molecule is absorbing most of the light.

### 3.2. Main Sensor Test

Data from the sensor evaluations were collected using Thorlabs OSA 2.9 software and then imported into Origin for plotting and analysis. The Thorlabs software only yields a plot of intensity versus wavelength; Origin was used to plot these values and to plot intensity values versus time.

The resulting graph consisted of the target, injection, and exposure curves, each for a time from 0 s (target), 1 s (initial exposure), 5 s to 150 s (one curve for each 5 s of exposure). The intensity values from each experiment were transferred to another sheet and plotted against these times to compare the light intensity decay and its progression from the time of initial exposure to the 150 s test.

The NH_3_ exposure experiments showed better results when the amount of pigment in the film was higher, while the HCl exposure values were good when the amount of pigment was moderate. The intensity versus time plots mentioned above required wavelength fixing for both NH_3_ and HCl exposure, due to the high intensity values shown for each wavelength value. Wavelength fixing was performed by observing the greatest changes in the intensity versus wavelength plots and comparing the distances between the reference and first exposure curves. The zones where the significant decrease in intensity was detected were 560.18 nm for NH_3_ and 600.06 nm for HCl.

**Figure 8:**
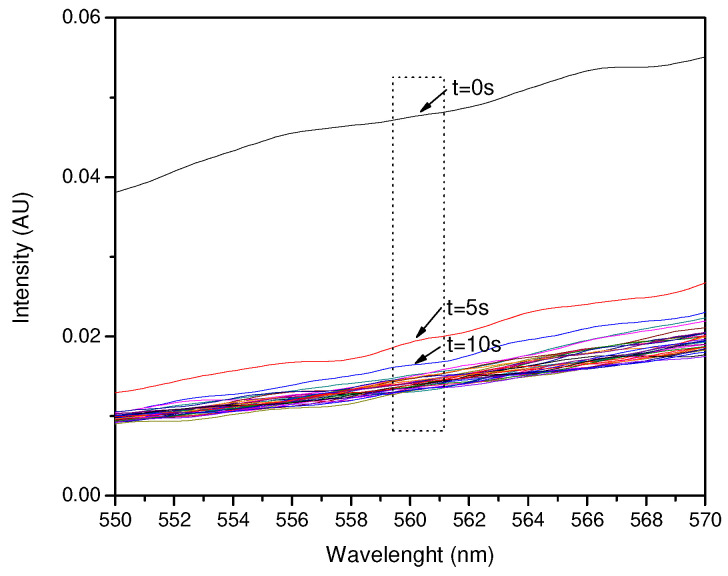
Spectral response of the exposure of 400 µL of the pigment against 100 µL of NH_3_ around 560 nm.

**Figure 9:**
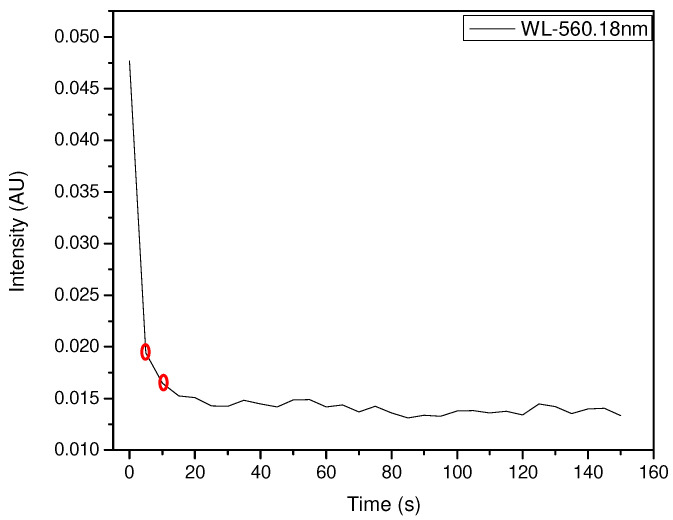
Intensity vs. time at 560.18 nm with early intensity decay (100 µL NH_3_ and 400 µL pigment).

The exposure of 100 µL of NH_3_ to a 400 µL pigment film is shown in Figure 8, and the decay of intensity versus time at 560.18 nm is shown in Figure 9. This test showed an early response at an exposure time of no more than 1 s with the intensity decaying from 0.0489 to 0.019, and then to 0.016 from 1 s to 10 s. The stability after 10 s shows variable values and can be predicted as a variation in environmental conditions other than the presence of NH_3_.

**Figure 10:**
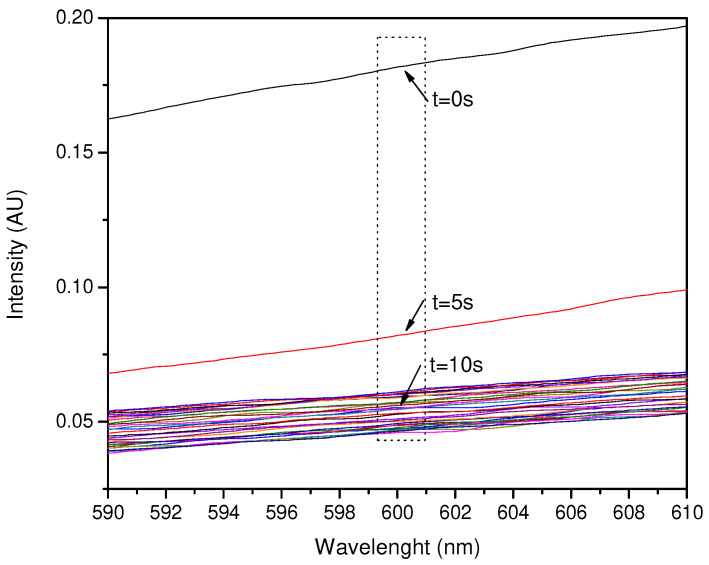
Spectral response of the exposure of 100 µL of the pigment against 100 µL of HCl around 600 nm.

**Figure 11:**
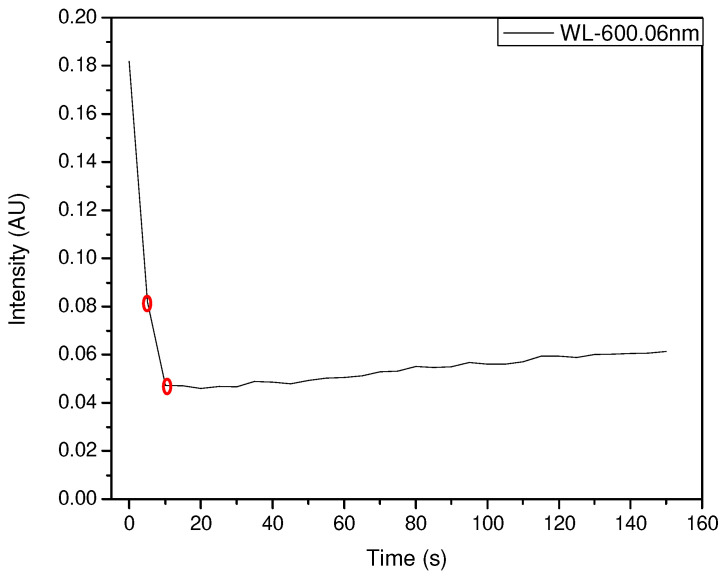
Intensity vs. time at 600.06 nm showing an early decay of the intensity (100 µL of HCl and 100 µL of pigment film).

In the case of HCl exposure, the best results were obtained by exposing 100 µL of HCl to a film of 100 µL of pigment. As with NH_3_, an early response of not more than 1 s was observed with a decrease in intensity from 0.18 to 0.08, and then to 0.045 after 5 s at a fixed 600.06 nm (Figure 10 and Figure 11).

The remaining experiments showed a late detection in the first 10 s of exposure; however, other alternatives can be used to speed up detection by using the substrate as a waveguide to measure the change in refractive index as a result of changes in physical properties [26,27]. The plot obtained from exposing 100 µL of pigment to 300 µL of NH_3_ showed instability with oscillating peaks. The case of 200 µL of pigment to 300 µL of HCl showed an unexpected increase in intensity within 1 s of exposure and then a decay of the same magnitude. As with these, other results were discarded to calculate detection limits from only the best repeatable results.

### 3.3. Light Absorption of Curcumin and Interaction with pH Value

The best results were obtained by exposing 100 µL of HCl to 100 µL of pigment, and 100 µL of NH_3_ to 400 µL of pigment, both with an early response of no more than 1 s. The mass of vapor exposed to the pigments at this time was calculated from the evaporation rates previously shown to be related to the internal volume of the spectrophotometric cells, and finally the detection limits of the sensor were estimated.

Equations (4) and (5) show the calculations of the mass of vapor emitted by the solutions in 1 s.

(4) $$m_{vNH_{3}}=\frac{\left(5.6\;mg)(1\;s\right)}{60\;s}=0.093\;mg=93\;\mu g$$

(5) $$m_{vHCl}=\frac{\left(2\;mg)(1\;s\right)}{60\;s}=0.033\;mg=33\;\mu g$$

The spectrophotometric cell volume was 3.2 mL. The calculated vapor mass contained in this volume was the minimum amount of gas capable of stimulating the pigment film to give a response to the sensor; the ratio of the vapor mass to the cell volume corresponds to the lower detection limit of the sensor (Equations (6) and (7)).

(6) $${DL}_{{NH}_{3}}=\frac{0.09\;mg}{3.2\;mL}=0.03\;ppm$$

(7) $${DL}_{HCl}=\frac{0.03\;mg}{3.2\;mL}=0.009\;ppm$$

The minimum detection limits of these gases in commercial equipment of the industry work in the ranges of 0–10 ppm for HCl and 0–100 ppm for NH_3_ [International Gas Detectors Trademark]. Phenolphthalein indicators can detect NH_3_ in aqueous solutions and can be wetted for detection in air with a detection range of 3–5 ppm [Bartovation Trademark PSP03V100], although other versions exist that have detection ranges of 0–100 ppm [Indigo Instruments Trademark 33810-Phe]; bromothymol blue is also used for the detection of NH_3_ and must likewise be wetted with distilled water to detect NH_3_ in air; however, the process is reversible and can be used again when exposed to CO_2_ [28] [Indigo Instruments]; in addition to these dyes, there are different detection techniques using polymers with sensitivities from 250 ppb [29] for HCl, and TiO_2_ thin films with a minimum sensitivity of 0.1 ppm [30] for HCl, nanocrystals with minimum detection ranges of 1–5 ppm [31] for HCl, and investigations with still exotic materials such as boron-doped carbon nanocones [32] for HCl; on the other hand, recent studies for detection of NH_3_ have experimented with photonic crystal fibers and graphene-assisted plasmon resonance [33], as well as the use of heterostructures with minimum detection limits of 0.155 ppm [34].

The detection limits of this proposal are good considering that the Occupational Safety and Health Administration standard defines a concentration of 5 ppm as hazardous to health and a concentration of 50 ppm as hazardous to life [35], making the valuable sensor in the industry to detect minimal leaks.

The detection limit values are a direct result of the experiment under controlled conditions; the sensitivity could be increased by direct deposition of the film over the optical fiber, but one of the main characteristics of the system would be lost; the easy replacement of the sensitive film, which makes the system stable and low cost of consumables.

## 4. Conclusions

A *C. longa* pigment-based fiber optic sensor was developed for the detection of HCl and NH_3_ vapors using pigment films, spectrophotometric cells, Thorlabs equipment, and software. Vapor detection was achieved using aqueous hydrochloric acid (37%) and ammonia (29%), injected through a septum into the cells where the pigment films were placed. The pigment of turmeric longa is composed mainly of curcumin, which gives it an intense orange-yellow color. This molecule absorbs light differently when its environment is acidic, neutral, or primary, so it presents an orange color in its neutral state, an intense red when it is basic, and dark brown or black in an acidic environment. When exposed to HCl (pH 1), the response changed to dark brown and black, causing a decrease in the intensity of light passing through the film and then the fiber. In this case, the sensor showed an early response of no more than 1 s on a 100 µL (2.3 mg) pigment film, and a lower detection limit of 0.009 ppm at 600.06 nm. When exposed to NH_3_, the sensor performed similarly, showing an early response of no more than 1 s with a shift to intense dark red. A lower detection limit of 0.03 ppm at 560.18 nm was determined with a 400 µL (9.2 mg) pigment film.

## Data Availability

Data and more details about the prototype could be requested by email to correspondence authors.
